# Albinism and Primary Immunodeficiency in Infants: A Case Study of Griscelli Syndrome

**DOI:** 10.7759/cureus.62178

**Published:** 2024-06-11

**Authors:** Nadia Bouhafs, Chaimae N'joumi, Aziza Elouali, Abdeladim Babakhouya, Maria Rkain, Noufissa Benajiba

**Affiliations:** 1 Department of Pediatrics, Centre Hospitalier Universitaire Mohammed VI Oujda, Oujda, MAR; 2 Department of Pediatrics, Faculty of Medicine and Pharmacy of Oujda, Mohammed I University, Oujda, MAR; 3 Department of Pediatric Gastroenterology, Centre Hospitalier Universitaire Mohammed VI Oujda, Oujda, MAR; 4 Department of Pediatric Hematology, Centre Hospitalier Universitaire Mohammed VI Oujda, Oujda, MAR

**Keywords:** microscopic examination, griscelli syndrome, hemophagocytic syndrome, immunodeficiency, albinism

## Abstract

Griscelli syndrome (GS) type II is a rare hereditary disorder characterized by partial albinism, immunodeficiency, and the subsequent development of hemophagocytic syndrome (HPS). Herein, we present a case involving a four-month-old infant admitted to our facility due to a prolonged fever complicated by HPS. The diagnosis of GS type 2 was established based on a constellation of clinical and laboratory findings: consanguinity, familial history of early infectious fatalities, ocular-cutaneous hypopigmentation, characteristic silvery hair sheen, onset of HPS, and notably, the pathognomonic appearance upon microscopic examination of a hair sample. The absence of giant granules within nucleated cells helped exclude Chediak-Higashi syndrome.

## Introduction

Albinism comprises a spectrum of genetic disorders marked by congenital deficiency or absence of melanin synthesis. While typically presenting in isolation, it may occasionally manifest alongside systemic anomalies, at times jeopardizing life prognosis (coagulation abnormalities, immunodeficiency), referred to as syndromic albinism.

Within this spectrum are Hermansky-Pudlak syndrome and Chediak-Higashi syndrome; the latter is characterized by impaired melanosome formation in melanocytes, while the exceedingly rare Griscelli-Prunieras syndrome is also identified [1].

In 1978, Griscelli initially described Griscelli syndrome (GS) as a rare and often fatal condition featuring partial albinism, variable cellular immunodeficiency, recurrent fever episodes, hepatosplenomegaly, and cytopenia [2]. Differential diagnosis includes syndromes such as Chediak-Higashi syndrome, Hermansky-Pudlak syndrome type 2, and p14 deficiency, which may present with both albinism and immunodeficiency.

This report details a case of a four-month-old infant with GS type 2, aiming to contribute to the scientific understanding of this complex syndrome.

## Case presentation

A male infant, aged four months, was admitted to the pediatric ward for evaluation of prolonged fever and chronic diarrhea persisting for one month prior to admission. Notable medical history included second-degree consanguinity and two sibling deaths in infancy. The first occurred at four months, while the other at one year of age, both in the context of prolonged fever and sepsis.

**Figure 1:**
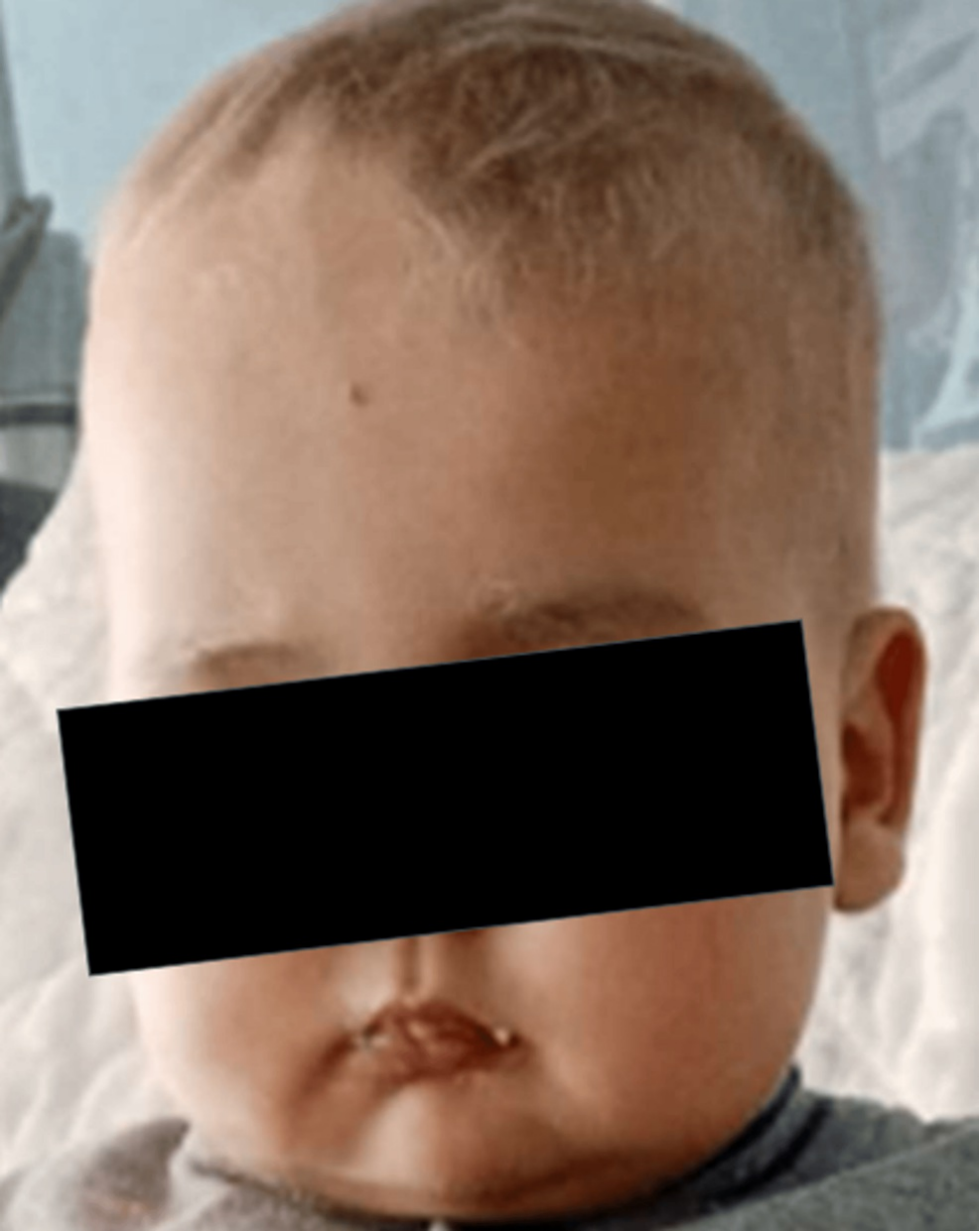
Photograph of our patient showing silver-gray hair and eyebrows.

Clinical examination revealed a febrile, pale, eutrophic infant, exhibiting abdominal distension with hepatomegaly extending 3 cm beyond the costal margin. Cutaneous examination demonstrated a lighter skin tone compared to the parents, with distinctive hair and eyebrow coloration characterized by a golden hue and silver sheen (Figure 1), a trait also observed in the deceased siblings.

Laboratory findings revealed normochromic normocytic non-regenerative anemia, thrombocytopenia, neutropenia, lymphopenia, hypertriglyceridemia, hypofibrinogenemia, and moderate hepatic cytolysis, with ferritin levels exceeding 2000 µg/L (Table 1). Given this presentation of febrile pancytopenia alongside hemophagocytic lymphohistiocytosis (HLH) features, bone marrow examination confirmed hemophagocytic activity, supporting a diagnosis of HLH flare-up according to Henter's criteria [3]. Given the age, family history, and especially albinism, primary HLH diagnosis was considered, particularly Chediak-Higashi syndrome or GS type 2.

**Table 1:** Biological results of our patient upon admission. SGOT: serum glutamic oxaloacetic transaminase; SGPT: serum glutamic pyruvic transaminase.

Parameters	Results	Reference value
Hemoglobin (g/dl)	6.2	9.5-13.5
Neutrophils (E/mm3)	1340	1500-7000
Lymphocytes (E/mm3)	1270	3500-16000
Platelets (E/mm3)	90000	1500-4000
Triglycerides (g/L)	4.45	<0.75
Fibrinogen (g/L)	0.7	1.5-3.5
Ferritin (ug/L)	2100	40.220
SGOT (UI/L)	75	8-30
SGPT (UI/L)	80	8-35

**Figure 2:**
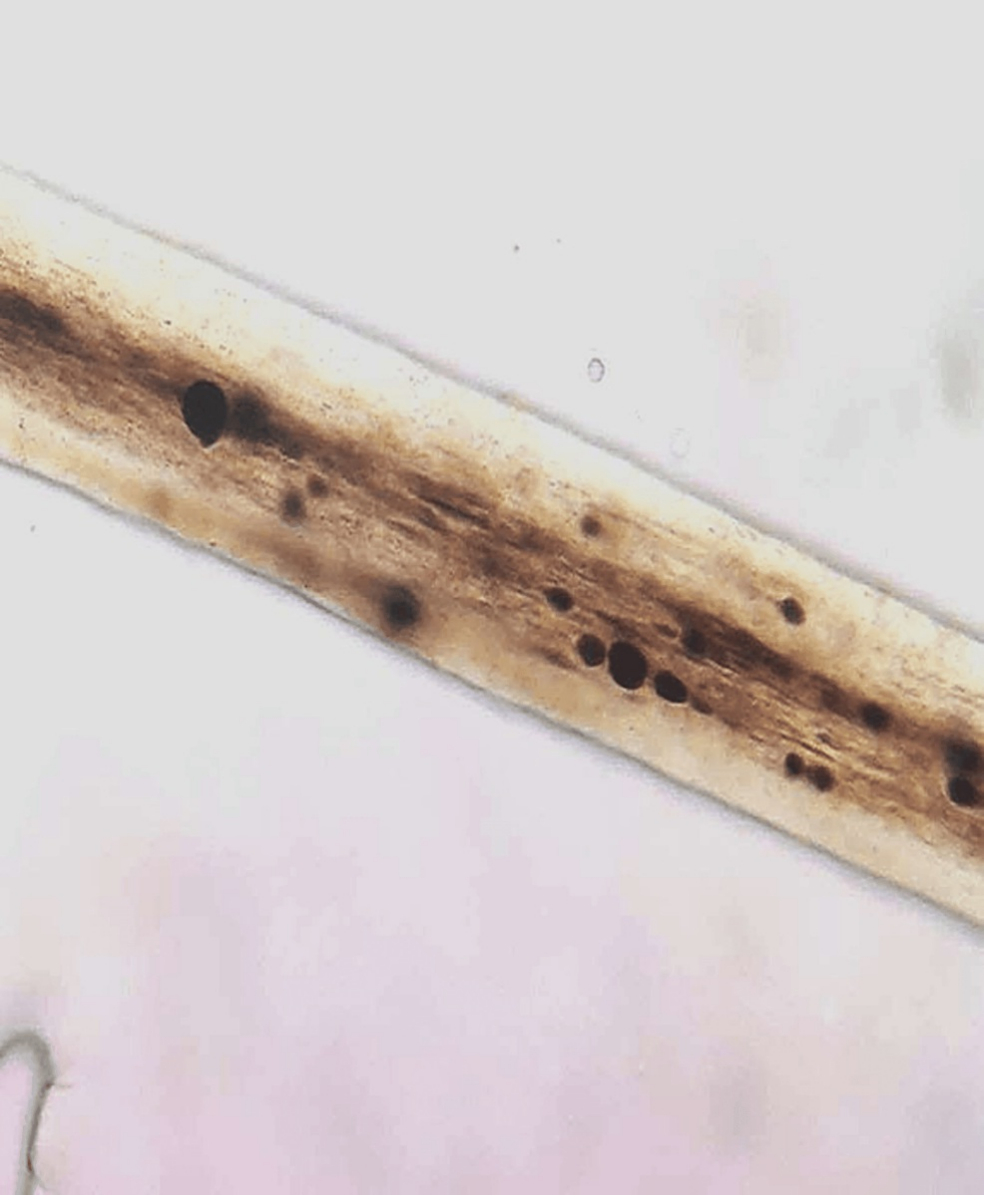
Histological study of hair under an optical microscope showing the presence of large granules distributed irregularly.

Accordingly, a comprehensive immune deficiency assessment was conducted, revealing reduced lymphocyte subpopulations: decreased CD3, CD4, CD19, and CD8, while human leukocyte antigen (HLA)-DR expression and perforin expression were normal. Hair examination under optical microscopy (Figure 2) and peripheral blood smear confirmed GS, with the absence of intracytoplasmic giant granules excluding the closely related Chediak-Higashi syndrome. The genetic study was not conducted due to a lack of resources.

The patient initially received corticosteroid therapy with cyclosporine according to the HLH-2004 protocol, showing a favorable initial response, with no activation episodes until the 26th week of treatment, when the patient presented with local neurological activation and later died at the age of 18 months in the context of sepsis.

## Discussion

GS was first described in 1978 by Griscelli and Siccardi in France [2]. It is a rare autosomal recessive genetic disorder caused by mutations in genes encoding the melanosome transport complex. The syndrome is characterized by albinism with silvery hair sheen and cutaneous hypopigmentation, which may be associated with neurological involvement (type I), immunodeficiency (type II), or isolated presentation (type III) [4].

Predominantly reported in Mediterranean regions, notably Turkey, with a few documented cases from India in 2004 [4], the global incidence of GS as of August 2023, stands at an estimated 160 cases [5]. Manifestations typically arise in early childhood, between four months and seven years of age, affecting both sexes.

GS is subcategorized into three types due to clinical and genetic variations. These variations are caused by mutations in three genes located on the 15q21 region, i.e., MYO5A, RAB27A, and MLPH, giving rise to three distinct clinical forms, respectively, GS1, GS2, and GS3, with albinism as a common feature [6]. GS2 is the most prevalent phenotype, while GS3 is the least observed [7].

GS type 1 is attributed to mutations in the MYO5A gene. In addition to ocular-cutaneous hypopigmentation, it presents with various neurological impairments, including severe and early psychomotor delay, intellectual disability, and epilepsy seizures. Elejalde syndrome is now considered part of GS1 by most authors. Corticosteroids and antiepileptic drugs generally fail to prevent early death caused by severe neurological involvement. To date, no curative treatment is available [8,9]. GS type 3 manifests solely as cutaneous involvement devoid of neurological or immunological sequelae, often leading to delayed diagnosis. Overall survival is similar to the general population, and no treatment is required.

On the other hand, GS2 results from mutations in the RAB27A gene. The RAB27A protein specifically regulates the secretion of cytotoxic granules. RAB27A abnormalities lead to neutropenia and dysfunction of natural killer (NK) cells, resulting in immunodeficiency and abnormal cytotoxic response that may culminate in uncontrolled lymphocyte and macrophage activation, known as hemophagocytic lymphohistiocytosis syndrome or HLH [9]. HLH is an inevitable complication of GS type II in the absence of bone marrow transplantation.

Patients diagnosed with GS2 may exhibit neurological symptoms that pose challenges in distinguishing between GS1 and GS2. In GS1, primary neurological impairments stem from the pivotal role of myosin Va in neuronal development, neuropeptide exocytosis, and synaptic plasticity, whereas in GS2, neurological manifestations result from lymphohistiocytic hemophagocytosis (HLH). Since RAB27A is not expressed in nerve cells, neurological alterations in GS2 are secondary to lymphohistiocytic infiltration of the central nervous system (CNS) [8].

Neurological manifestations in GS2 vary widely and may include seizures, hearing impairment, language or vision disorders, ataxia, tremors, and signs of elevated intracranial pressure. Brain imaging typically reveals hyperintense lesions, occasionally resembling demyelinating diseases.

The differentiation between GS1 and GS2 is crucial, particularly for timely diagnosis, as hematopoietic stem cell transplantation (HSCT) can improve neurological outcomes in GS2 but not in GS1.

The diagnosis of GS type 2 is established by microscopic examination of the hair, revealing pigmentary dilution with clusters of pigment irregularly distributed in the hair shaft, as well as a polychromatic appearance under polarized light. Detection of a homozygous mutation in the RAB27A gene further confirms the diagnosis. Prenatal screening is recommended for couples with a family history of GS [5].

Chediak-Higashi syndrome stands as the primary differential diagnosis. It arises from mutations in the LYST gene responsible for encoding the CHS protein. Clinically, it manifests with concurrent partial albinism and immune deficiency. Biologically, it is marked by the presence of giant intracytoplasmic granules in polymorphonuclear cells and azurophilic granules in lymphocytes, features absent in GS type 2 [10].

Regarding the therapeutic management of GS2, chemotherapy (VP16) or more recently, anti-lymphocyte serum and cyclosporine have achieved short-term remission. Intrathecal methotrexate injections have shown efficacy regarding cerebral involvement. Currently, bone marrow transplantation stands out as the sole treatment option capable of achieving complete and sustained remission.

## Conclusions

GS is a rare disorder suspected based on the unique appearance of hair under polarized light, confirmed through molecular biology. The prognosis is severe in the absence of bone marrow transplantation and early detection and intervention; however, they significantly improve outcomes, especially for GS type 2.
